# Supplementary material for: Changes in Health Behaviors, Mental and Physical Health among Older Adults under Severe Lockdown Restrictions during the COVID-19 Pandemic in Spain
Source: Int J Environ Res Public Health. 2021 Jul 1;18(13):7067. doi: 10.3390/ijerph18137067 (PMC8297096; doi:10.3390/ijerph18137067)
Supplement: Supplementary file 1 [file ijerph-18-07067-s001.zip › ijerph-1267253-SI.pdf]

Table S1: Prevalence or mean (SD) for the main sociodemographic, lifestyle and health-related characteristics of older adults at the COVID-19 pre-confinement period (t0) and the State of Alarm (t1)†

	ENRICA (n=1323)		*ES* (n=464)		*TSHA* (n=829)		**Exernet** (n=425)	
	**t0**	**t1**	**t0**	**t1**	**t0**	**t1**	**t0**	**t1**
**Socio-demographic variables**								
**Age;** $\bar{x}$ (SD)	73.7(4.3)	75.4(4.3)	69.9(8.0)	69.9(8.0)	76.8(5.5)	81.5(5.6)	77.8(4.5)	81.3(4.5)
**Male**; %	50.5		42.9		39.8		21.2	
**University**; %	22.3		16.4		8.2		8.0	
**Civil status**; %								
Married	65.2		64.2		68.0		57.4	
Single	7.0		4.1		4.2		4.0	
Divorced	5.9		10.3		2.1		2.6	
Widowed	21.9		21.3		25.7		35.9	
**Living alone**; %	23.6	24.1	21.6	18.5	21.2	25.2	34.1	35.8
**No daily contact** with family/friends; %	12.9	7.3	47.9	20.8	26.5	10.6		
**Feeling lonely (1-5 scale)**; $\bar{x}$ (SD)	1.6 (0.9)	1.4 (0.9)	1.5 (1.1)					
**Housing conditions**								
**No outdoor views**; %	8.2		9.1		4.3		9.9	
**No terrace/balcony**; %	25.7		32.8		37.0		24.0	
**No private garden/yard**; %	86.6		72.8		41.9		72.2	
**Lack of internet**; %	20.5		23.1		54.9		63.3	
**Too much noise**; %	2.3		4.7		3.1		3.3	
**Size of house (m^2)**; $\bar{x}$	95.6							
**Lifestyle-behaviours**								
**Smoking**; %								
Never smokers	51.1	52.1	54.6	50.0	69.9	71.7	79.5	89.9
Former smokers	39.4	40.9	28.9	34.5	23.1	24.5	16.7	9.4
Current smoker	8.9	7.0	16.4	15.5	6.8	3.7	1.7	0.7
**Exposure to SHS;** %	4.3	9.9						
**Alcohol intake**; %								
Not drinker	42.6	46.0	54.1	52.4	62.3	66.1		
Drinker, not daily	28.0	19.1	29.1	26.5	10.1	10.6		
Drinker, daily/almost daily	29.4	35.0	16.8	21.1	27.6	23.3		
**MEDAS index**; $\bar{x}$ (SD)	7.2 (1.7)	7.4 (1.7)	8.5 (1.8)	7.5 (1.7)	7.4 (1.7)	6.6 (1.8)	9.0 (2.0)	7.4 (1.6)
**Eating at a regular time; %**		94.7		-		96.8		96.5
**Eating more ultra-processed, %**		2.3		5.3		0.6		0.2
**Eating snacks**	45.6	37.3						
**Eating to calm anxiety**	13.2	8.1						

**rPA (Mets-h/week)**; x̄ (SD)	53.2 (24.9)	43.1 (27.3)	23.5 (37.5)	‡			81.8 (40.0)	36.0 (27.1)
**hPA Mets-h/week)**; x̄ (SD)	24.8 (19.6)	23.6 (21.3)					44.6 (34.4)	22.0 (22.5)
**PASE score**; x̄ (SD)					87.8 (44.0)	69.7 (52.6)		73.8 (60.0)
**BMI**								
Normoweight	29.0		27.8		14.8		19.5	
Overweight	48.9		43.1		42.0		46.1	
Obese	23.1		29.1		43.2		34.4	
**Weight;** § x̄ (SD)	72.2 (12.7)	71.8 (12.1)	73.8 (13.9)	73.6 (13.3)	73.4 (13.1)	71.7 (13.0)	69.3 (11.1)	67.9 (11.0)
**Total ST (h/d)**; x̄ (SD)	6.1 (2.7)	7.2 (3.2)	4.8 (2.5)	‡		6.7 (3.2)	4.3 (2.1)	6.8 (3.3)
**TV viewing time (h/d);** x̄ (SD)	3.5 (1.8)	4.0 (2.2)						
**Other screen time** (min/day); x̄ (SD)	36.5 (64.8)	82.0 (96.4)						
**Reading** (min/day)**; x̄ (SD)**	51.8 (58.5)	68.2 (74.7)						
**Listening to music (min/day); x̄ (SD)**	11.3 (33.6)	19.2 (49.6)						
**Sleep characteristics**								
**Hours of night-time sleep**; %								
Normal sleep	79.2	73.0	77.2	75.0	62.6	62.7	66.6	67.1
Short sleep (≤6 h)	16.3	18.2	11.9	16.4	18.1	18.5	25.4	14.4
Long sleep (≥9 h)	4.5	8.8	11.0	8.6	19.3	18.8	8.0	18.5
**Hours of day-time sleep**; %								
None	394	717					205	236
Short nap (≤30 min)	623	556					125	180
Long nap (30-60 min)	226	41					62	5
Very long nap (≥60min)	71	0					29	0
**Overall sleep quality**; %								
Very good	65.1	62.6	47.4	72.6			46.2	64.6
Good	10.8	11.2	41.8	11.4			23.0	6.1
Fair	21.8	22.0	9.9	13.7			23.2	25.1
Poor/very poor	2.3	4.2	0.9	2.3			7.7	4.3
**Poor sleep quality score;**‖ x̄ (SD)	1.7 (1.5)	1.8 (1.5)						
**Health-related variables**								
**Overall health**; x̄ (SD)								
SF-12, PCS	46.0 (11.0)	50.2 (8.6)						
SF-12, MCS	54.1 (9.6)	53.2 (8.5)						
WHODAS-12			9.4 (15.5)	11.9 (17.1)				
EQ-5D					0.9 (0.2)		0.8 (0.2)	
**Pain scale***; x̄ (SD)	0.9 (2.2)	1.4 (2.2)						
**Mobility limitations;** %	36.0							
**Negative ageing experience scale*;** x̄ (SD)	2.5 (1.0)							
**Cantril ladder***; x̄ (SD)	8.1 (1.8)							

**Low MMSE scores (<23)**; %	3.4				
**Week of state of alarm**; † range		7-11	10-15	8-11	7-11

ENRICA: Seniors-ENRICA-2. ES: Edad con Salud. TSH: Toledo Study for Healthy Ageing.

$\bar{x}$: Mean; SD: Standard deviation; MEDAS: Mediterranean Diet Assessment Score; rPA: Recreational physical activity; hPA: Household physical activity; BMI: Body Mass Index; ST: Sedentary time; PCS: Physical Component Score of the SF-12; MCS: Mental Component Score of the SF-12

†The Spanish Government declared the state of alarm on March 14th, meaning general lockdown for the entire country from March 15th. From May 4th (week 8) adults were allowed out to walk and exercise a maximum of 1 hour/dat during set time slots. The de-escalation phase was gradual during the following weeks, until the country started its "New Normality" phase on June 22nd (week 14)

‡ Differences between questionnaires did not allow for pre-post comparisons

§ Information at follow-up was only available in the subsample of participants that had weight themselves on the week of the telephone interview (n=1566)

‖ The score ranged 0 (best) to 7 (worse sleep). Participants who answered "sometimes" or "almost always" to four indicators of poor night-time sleep (i.e. difficulty falling asleep, awakening during night-time, early awakening with difficulty getting back to sleep, use of sleeping pills), two indicators of drowsiness (being so sleepy at daytime as to need a nap, not feeling rested in the morning), or with a score >10 in the Epworth Sleepiness Scale, received 1 point; their counterparts received 0 points

¶ Higher scores in the MCS and PCS of the SF-12 as well as on the EQ-5D, and lower scores in the WHODAS-12 are indicative of better health.

*The pain scale ranged 0 (best) to 7 (worse pain) and included information on frequency, intensity and number of pain sites. Individuals with sporadic (<2 time/day) and frequent (≥2 times/week) pain were assigned a score of 1 and 2, respectively; those with light, moderate and high intensity pain were given a score of 1,2 and 3; and those with 1-2 and ≥3 locations of pain a score of 1 and 2, respectively. The ageing experience scale ranged from 1 (very positive experience) to 5 (very negative experience). The Cantril ladder ranged from 1 (less satisfied) to 10 (more satisfied).

Table S2. Prospective association between baseline participant characteristics and changes in frequency of tobacco (n=194: 93 from *Seniors ENRICA-2*, 67 from *Edad con Salud*, 31 from *TSHA*, and 3 from *Exernet*) and alcohol (n=2616: 1323 from *Seniors ENRICA-2*, 464 from *Edad con Salud*, and 829 from *TSHA*) consumption between the COVID-19 pre-confinement period and the State of Alarm

	**Changes in frequency of tobacco consumption**				**Changes in frequency of alcohol consumption**				
	**n**	**Same frequency**	**Increased frequency**	**Decreased frequency**	**n**	**Same frequency**	**Increased frequency**	**Decreased frequency**	**Maintained non-drinking status**
		RRR(95%CI) n=99	RRR (95%CI) n=42	RRR (95%CI) n=53		RRR(95%CI) n=731	RRR (95%CI) n=400	RRR (95%CI) n=447	RRR (95%CI) n=1038
**Socio-demographic characteristics**									
**Age, yr**	194	Ref.	1.02 (0.92;1.13)	1.02 (0.92;1.13)	2616	Ref.	**1.03 (1.00;1.05)**	**1.03 (1.00;1.06)**	**1.03 (1.01;1.06)**
**Sex**									
Male	114	Ref.	Ref.	Ref.	1197	Ref.	Ref.	Ref.	Ref.
Female	80	Ref.	1.27 (0.45;3.58)	0.80 (0.31;2.09)	1419	Ref.	**1.55 (1.14;2.11)**	**1.95 (1.45;2.71)**	**4.65 (3.58;6.04)**
**Education**									
Primary or less	102	Ref.	Ref.	Ref.	1694	Ref.	Ref.	Ref.	Ref.
Secondary	49	Ref.	2.09 (0.67;6.57)	0.74 (0.26;2.16)	483	Ref.	1.03 (0.74;1.43)	1.01 (0.73;1.39)	**0.71 (0.53;0.95)**
University	43	Ref.	1.94 (0.58;6.54)	0.91 (0.29;2.86)	439	Ref.	0.89 (0.62;1.26)	1.03 (0.74;1.43)	**0.62 (0.45;0.85)** *
**Civil status**									
Married	122	Ref.	Ref.	Ref.	1725	Ref.	Ref.	Ref.	Ref
Never married	11	Ref.	0.46 (0.03;7.32)	3.87 (0.44;34.3)	146	Ref.	0.67 (0.33;1.36)	1.07 (0.60;1.92)	1.05 (0.62;1.79)
Divorced	20	Ref.	0.49 (0.07;3.35)	0.26 (0.04;1.90)	143	Ref.	0.73 (0.37;1.43)	1.10 (0.60;2.00)	1.22 (0.72;2.06)
Widowed	41	Ref.	0.74 (0.15;3.51)	**0.19 (0.04;0.89)**	602	Ref.	1.25 (0.78;1.99)	0.90 (0.56;1.42)	1.33 (0.90;1.96)
**Living alone**									
No	151	Ref.	Ref.	Ref.	2028	Ref.		Ref.	Ref.
Yes	43	Ref.	1.74 (0.41;7.35)	1.33 (0.26;6.71)	588	Ref.	1.06 (0.65;1.71)	1.30 (0.82;2.07)	0.06 (0.65;1.43)

**Daily contact family/friends**									
Yes	137	Ref.	Ref.	Ref.	1989	Ref.	Ref.	Ref	Ref.
No	54	Ref.	1.01 (0.34;2.93)	1.83 (0.71;4.72)	623	Ref.	0.88 (0.63;1.23)	0.86 (0.63;1.19)	1.17 (0.89; 1.54)
**Feeling lonely (1-5 scale)†**	159	Ref.	1.05 (0.65;1.69)	0.96 (0.57;1.63)	1776	Ref.	1.15 (0.98;1.36)	1.03 (0.87;1.23)	1.10 (0.96;1.28)
**Housing conditions**									
**Lack of outdoor views**									
No	186	Ref.	Ref.	Ref.	186	Ref.	Ref.	Ref.	Ref.
Yes	8	Ref.	0.74 (0.06;9.77)	3.21 (0.42;24.3)	2430	Ref.	**1.75 (1.03;3.10)**	**2.23 (1.35;3.68)**	**2.30 (1.44;3.66)**
**Lack of terrace/balcony**									
No	148	Ref.	Ref.	Ref.	1817	Ref.	Ref.	Ref	Ref.
Yes	46	Ref.	**0.34 (0.10;1.00)**	0.95 (0.32;2.77)	799	Ref.	**0.69 (0.51;0.93)**	1.18 (0.90;1.55)	1.25 (0.99;1.59)
**Lack of garden/yard**									
No	39	Ref.	Ref.	Ref.	789	Ref.	Ref.	Ref.	Ref.
Yes	144	Ref.	2.76 (0.77;9.88)	0.81 (0.29;2.28)	1830	Ref.	0.90 (0.66;1.23)	1.14 (0.84;1.55)	**1.33 (1.02;1.74)**
**Internet access**									
Yes	151	Ref.	Ref.	Ref.	1783	Ref.	Ref.	Ref.	Ref.
No	43	Ref.	1.92 (0.56;6.66)	**0.31 (0.00;1.04)**	833	Ref.	1.21 (0.88;1.68)	1.10 (0.80;1.51)	**1.61 (1.23;2.11)**
**Too much noise**									
No	188	Ref.	Ref.	Ref.	2537	Ref.	Ref.	Ref.	Ref.
Yes	6	Ref.	1.45 (0.09;23.6)	1.97 (0.22;17.4)	79	Ref.	0.72 (0.32;1.64)	1.13 (0.56;2.27)	0.89 (0.47;1.66)
**Lifestyle-behaviours**									
**Smoking**									
Never smokers	-	Ref.	-	-	1507	Ref.	Ref.	Ref.	Ref.
Former smokers	-	Ref.	-	-	853	Ref.	**0.59 (0.44;0.80)**	0.94 (0.71;1.25)	**0.59 (0.45;0.76)**

Smoker	-	Ref.	-	-	247	Ref.	0.88 (0.57;1.35)	1.14 (0.75;1.73)	**0.63 (0.43;0.94)**
**MEDAS**	186	Ref.	1.17 (0.54;6.92)	1.11 (0.87;1.40)	2616	Ref.	**0.93 (0.86;0.99**)	0.96 (0.89;1.03)	**0.92 (0.86;0.98)**
**PA** (cohort-specific quartiles)									
1st quartile (less activity)	56	Ref.	Ref.	Ref.	676	Ref.	Ref.	Ref.	Ref.
2nd quartile	40	Ref.	0.40 (0.11;1.45)	1.08 (0.31;3.71)	667	Ref.	1.02 (0.72;1.44)	1.21 (0.85;1.72)	1.00 (0.73;1.37)
3rd quartile	49	Ref.	0.54 (0.16;1.85)	1.43 (0.46;4.43)	665	Ref.	0.79 (0.55;1.14)	1.08 (0.76;1.54)	0.86 (0.62;1.18)
4th quartile	49	Ref.	0.99 (0.30;3.28)	1.61 (0.50;5.20)	608	Ref.	0.71 (0.49;1.04)	0.97 (0.67;1.40)	1.03 (0.74;1.41)
**BMI**									
Normoweight	73	Ref.	Ref.	Ref.	601	Ref.	Ref.	Ref.	Ref.
Overweight	83	Ref.	0.82 (0.30;2.24)	**2.60 (1.00;6.77)**	1216	Ref.	1.19 (0.86;1.65)	1.05 (0.77;1.42)	1.07 (0.82;1.42)
Obese	38	Ref.	0.73 (0.20;2.59)	**2.97** (0.97;9.67)	799	Ref.	1.26 (0.87;1.84)	1.15 (0.81;1.65)	1.24 (0.90;1.70)
**ST** (cohort-specific quartiles)†									
1st quartile (less time)	47	Ref.	Ref.	Ref.	504	Ref.	Ref.	Ref.	Ref
2nd quartile	21	Ref.	**8.21 (1.54;43.7)**	0.24 (0.02;2.54)	407	Ref.	0.89 (0.60;1.34)	1.27 (0.84;1.92)	0.87 (0.61;1.25)
3rd quartile	48	Ref.	2.21 (0.57;8.48)	1.37 (0.38;4.97)	444	Ref.	**0.61 (0.40;0.92)**	1.00 (0.66;1.51)	0.84 (0.59;1.20)
4th quartile	47	Ref.	1.88 (0.45;7.82)	3.00 (0.72;12.3)	432	Ref.	0.98 (0.66;1.46)	1.32 (0.87;2.00)	0.79 (0.54;1.14)
**Sleep characteristics**									
**Hours of night-time sleep**									
Normal sleep	144	Ref.	Ref.	Ref.	1925	Ref.	Ref.	Ref.	Ref.

Short sleep (≤6 h)	28	Ref.	1.93 (0.54;6.92)	0.39 (0.10;1.53)	421	Ref.	1.19 (0.84;1.68)	0.99 (0.70;1.41)	1.11 (0.47;1.66)	
Long sleep (≥9 h)	22	Ref.	1.14 (0.26;4.97)	0.86 (0.21;3.48)	270	Ref.	1.42 (0.88;2.29)	1.39 (0.88;2.18)	**1.77 (1.23;2.11)**	
**Hours of day-time sleep‡**										
None	27	Ref.	Ref.	Ref.	395	Ref.	Ref.	Ref.	Ref.	
Short nap (≤30 min)	40	Ref.	0.18 (0.02;1.44)	1.47 (0.16;13.4)	625	Ref.	0.88 (0.59;1.32)	0.90 (0.61;1.33)	0.81 (0.57;1.16)	
Long nap (30-60 min)	18	Ref.	0.32 (0.03;3.53)	1.53 (0.12;19.1)	229	Ref.	1.18 (0.71;1.93)	0.69 (0.41;1.18)	0.79 (0.49;1.26)	
Very long nap (≥60min)	11	Ref.	0.29 (0.02;4.75)	0.99 (0.06;15.3)	71	Ref.	**0.39 (0.16;0.96)**	1.04 (0.50;2.16)	0.56 (0.26;1.19)	
**Overall sleep quality†**										
Very good	100	Ref.	0.73 (0.16;3.40)	0.64 (0.19;2.22)	337	Ref.	0.96 (0.64;1.45)	1.07 (0.71;1.61)	0.96 (0.64;1.45)	
Good	35	Ref.	Ref.	Ref,	1081	Ref.	Ref.	Ref.	Ref.	
Fair	280	Ref.	**6.16 (1.28;29.6)**	0.66 (0.11;4.09)	335	Ref.	**0.65 (0.43;0.98)**	0.78 (0.51;1.18)	**0.64 (0.43;0.98)**	
Poor/very poor		Ref.	-	-	34	Ref.	1.24 (0.34;4.48)	**3.73 (1.15;12.1)**	1.24 (0.34;4.48)	
**Health variables**										
**Overall health**										
1st quartile (worst)	33	Ref.	Ref.	Ref.	512	Ref.	Ref.	Ref.	Ref.	
2nd quartile	51	Ref.	3.71 (0.69;20.0)	1.23 (0.30;5.01)	486	Ref.	1.09 (0.71;1.69)	1.09 (0.70;1.71)	1.05 (0.73;1.53)	
3rd quartile	27	Ref.	1.53 (0.21;11.1)	0.52 (0.10;2.81)	523	Ref.	0.90 (0.58;1.38)	1.05 (0.69;1.63)	**0.61 (0.42;0.89)**	
4th quartile (best)	83	Ref.	1.60 (0.29;8.99)	0.30 (0.07;1.25)	1095	Ref.	0.82 (0.53;1.27)	1.06 (0.69;1.64)	**0.51 (0.42;0.88)**	*
**Pain scale‡**	77	Ref.	1.10 (0.56;2.15)	0.67 (0.32;1.41)	1049	Ref.	0.98 (0.88;1.09)	0.95 (0.85;1.05)	0.98 (0.89;1.08)	
**Chronic morbidities**										

Diabetes	28	Ref.	1.70 (0.51;5.70)	0.40 (0.11;1.52)	471	Ref.	0.95 (0.67;1.34)	0.90 (0.64;1.27)	1.30 (0.98;1.74)
Hypertension	91	Ref.	0.51 (0.21;1.25)	0.72 (0.32;1.64)	1480	Ref.	1.28 (0.98;1.67)	1.11 (0.86;1.44)	1.02 (0.81;1.29)
CVD	22	Ref.	5.23 (0.70;39.3)	1.88 (0.30;11.9)	199	Ref.	0.80 (0.47;1.34)	1.10 (0.68;1.78)	0.92 (0.60;1.41)
Cancer	15	Ref.	1.40 (0.07;2.38)	**0.05 (0.00;0.46)**	188	Ref.	1.03 (0.64;1.68)	0.89 (0.55;1.44)	1.02 (0.67; 1.55)
Osteomuscular disease	67	Ref.	1.03 (0.37;2.88)	0.39 (0.15;1.06)	1041	Ref.	0.89 (0.67;1.17)	0.86 (0.66;1.13)	0.79 (0.62;1.02)
Depression	26	Ref.	0.78 (0.17;3.63)	1.00 (0.23;4.32)	311	Ref.	1.20 (0.75;1.91)	1.15 (0.73;1.81)	1.44 (0.97;2.11)
**Mobility limitations‡**	29	Ref.	3.57 (0.41;31.3)	0.29 (0.03;2.70)	476	Ref.	0.78 (0.50;1.22)	0.95 (0.62;1.47)	1.04 (0.71;1.54)
**Negative ageing experience scale ‡**	92	Ref.	0.91 (0.44;1.88)	0.52 (0.24;1.12)	1313	Ref.	0.99 (0.83;1.19)	1.09 (0.91;1.30)	0.97 (0.82;1.15)
**Cantril ladder§**	158	Ref.	1.28 (0.96;1.70)	1.08 (0.82;1.43)	1770	Ref.	1.03 (0.94;1.12)	0.96 (0.88;1.04)	1.02 (0.94;1.10)
**Low MMSE scores (<23)§**	6	Ref.	0.14 (0.00;3.29)	0.74 (0.04;14.6)	77	Ref.	1.07 (0.41;3.81)	1.99 (0.80;4.79)	1.68 (0.78;3.63)
**Years since baseline examination**	194	Ref.	2.20 (0.60;8.03)	3.31 (0.97;11.3)	2616	Ref.	0.99 (0.75;1.31)	0.93 (0.72;1.21)	1.06 (0.84;1.34)
**Week of the state of alarm**	194	Ref.	0.92 (0.60;1.41)	0.97 (0.63;1.49)	2616	Ref.	0.96 (0.84;1.10)	1.01 (0.89;1.15)	0.99 (0.88;1.11)

OR: Odds Ratio. 95%CI: 95% Confidence interval.; MEDAS: Mediterranean Diet Assessment Score; PA: Physical activity; BMI: Body Mass index; ST: Sedentary time; MMSE: Mini Mental State Examination

Note: Figures highlighted in bold show a statistically significant association.

† Information not available in the *TSHA* cohort; ‡ Information only available in the *Seniors ENRICA-2* cohort; § Information only available in the *Seniors ENRICA-2* and *Edad con Salud* cohorts

All models were adjusted for baseline age, sex (men or women), educational level (primary or less, secondary, or university), civil status (married, widowed, never married, divorced), smoking status (never, former, or current), adherence to the Mediterranean diet (MEDAS score), body mass index (normoweight, overweight, or obese), physical activity (quartiles), hours of sleep at night (normal, short sleep, long sleep), chronic comorbidities (hypertension, diabetes, cardiovascular disease, cancer, osteo-muscular disease and depression), overall health (SF-12, WHODAS-12 o EQ5D), time since last follow-up visit, cohort of study and week of the state of alarm.

* p-value for trend <0.05

Table S3. Prospective association between participant characteristics at baseline and changes in adherence to the MEDAS score between the COVID-19 pre-confinement period and the State of Alarm (n=3041: 1323 from *Seniors ENRICA-2*, 464 from *Edad con Salud*, 829 from *TSHA*, and 425 from *EXERNET*).

		Changes in MEDAS			
	n	No changes	Decreased ≥1 point	Increased ≥1 point	Mean changes
			OR (95%CI)	OR (95%CI)	$\bar{x}$ (95%CI)
		n=162	n=949	n=470	n=3045
**Socio-demographic characteristics**					
**Age, yr**	3041	Ref.	**1.02 (1.00; 1.04)**	0.98 (0.96; 1.01)	**-0.02 (-0.03; -0.01)**
**Sex**					
Male	1287	Ref.	1.00	Ref.	Ref.
Female	1754	Ref.	0.93 (0.73; 1.19)	0.89 (0.68; 1.18)	0.11 (-0.03; 0.25)
**Education**					
Primary or less	2040	Ref.	Ref.	Ref.	Ref.
Secondary	528	Ref.	0.94 (0.72; 1.22)	1.02 (0.75; 1.38)	0.09 (-0.06; 0.25)
University	473	Ref.	**0.73 (0.54; 0.99)** *	0.89 (0.64; 1.24)	**0.13** (-0.04; 0.30)
**Civil status**					
Married	1969	Ref.	1.00	1.00	Ref.
Never married	163	Ref.	0.99 (0.61; 1.61)	0.76 (0.42; 1.37)	-0.02 (-0.30; 0.27)
Divorced	154	Ref.	1.11 (0.67; 1.83)	0.98 (0.55; 1.73)	-0.15 (-0.44; 0.14)
Widowed	755	Ref.	1.13 (0.81; 1.56)	1.05 (0.72; 1.55)	**-0.20 (-0.39; -0.01)**
**Living alone**					
No	2308	Ref.	Ref.	Ref.	Ref.
Yes	733	Ref.	1.07 (0.77; 1.49)	0.91 (0.61; 1.35)	0.04 (-0.15; 0.23)
**Daily contact with family/friends other than cohabitants**†					
Yes	1989	Ref.	Ref.	Ref.	Ref.
No	623	Ref.	1.26 (0.97; 1.63)	0.79 (0.57; 1.10)	**-0.26 (-0.42; -0.10)**
**Feeling lonely (1-5 scale)**‡	1776	Ref.	1.12 (0.97; 1.29)	0.94 (0.80;1.10)	**-0.08 (-0.16; -0.01)**
**Housing conditions**					
**Lack of outdoor views**					
No	2816	Ref.	Ref.	Ref.	Ref.
Yes	228	Ref.	**1.53 (1.05; 2.21)**	0.90 (0.58; 1.40)	**-0.29 (-0.50; -0.07)**
**Lack of terrace/balcony**					
No	2140	Ref.	Ref.	Ref.	Ref.

Yes	904	Ref.	1.10 (0.88; 1.36)	1.09 (0.85; 1.41)	-0.08 (-0.21; 0.04)
**Lack of private garden/yard**					
No	907	Ref.	Ref.	Ref.	**Ref.**
Yes	2137	Ref.	1.09 (0.87; 1.37)	0.85 (0.64; 1.12)	-0.05 (-0.19; 0.08)
**Internet access**					
Yes	1939	Ref.	Ref.	**Ref.**	**Ref.**
No	1105	Ref.	1.10 (0.88; 1.38)	**0.74 (0.56; 0.98)**	**-0.16 (-0.29; -0.03)**
**Too much noise**					
No	2951	Ref.	Ref	Ref.	**Ref.**
Yes	93	Ref.	1.52 (0.85; 2.68)	0.99 (0.55; 1.87)	-0.23 (-0.56; 0.09)
**Lifestyle-behaviours**					
**Smoking**					
Never smokers	1863	Ref.	Ref.	Ref.	Ref.
Former smokers	924	Ref.	0.88 (0.68; 1.14)	0.99 (0.75; 1.29)	0.09 (-0.05; 0.24)
Smoker	254	Ref.	1.35 (0.92; 1.99)	1.05 (0.71; 1.57)	-0.14 (-0.37; 0.07)
**Alcohol intake**†					
Not drinker	1330	Ref.	Ref.	Ref.	Ref.
Drinker, not daily	590	Ref.	0.77 (0.58; 1.03)	0.85 (0.62; 1.16)	0.10 (-0.06; 0.26)
Drinker, daily or almost daily	696	Ref.	**0.63 (0.47; 0.83)**	1.03 (0.76; 1.39)	**0.22 (0.06; 0.38)**
**PA (cohort-specific quartiles)**					
1st quartile (less activity)	785	Ref.	Ref.	Ref.	Ref.
2nd quartile	771	Ref.	1.16 (0.88; 1.52)	0.74 (0.54; 1.02)	-0.16 (-0.31; 0.00)
3rd quartile	784	Ref.	0.92 (0.69; 1.21)	0.89 (0.64; 1.22)	0.02 (-0.14; 0.18)
4th quartile	701	Ref.	0.79 (0.59; 1.06) *	0.83 (0.60; 1.16)	0.08 (-0.09; 0.25)
**BMI**					
Normoweight	705	Ref.	Ref.	Ref.	Ref.
Overweight	1391	Ref.	0.86 (0.67; 1.11)	0.99 (0.74; 1.32)	-0.02 (-0.17; 0.12)
Obese	945	Ref.	1.04 (0.78; 1.38)	1.05 (0.75; 1.46)	-0.12 (-0.29; 0.04)
**ST (cohort-specific quartiles)**§					
1st quartile (less time)	635	Ref.	Ref.	Ref.	Ref.
2nd quartile	505	Ref.	1.03 (0.75; 1.42)	1.05 (0.72; 1.55)	-0.06 (-0.24; 0.12)
3rd quartile	554	Ref.	1.19 (0.87; 1.63)	1.17 (0.81; 1.69)	-0.05 (-0.23; 0.12)
4th quartile	518	Ref.	0.80 (0.57; 1.12)	0.95 (0.65; 1.38)	0.05 (-0.13; 0.23)
**Sleep characteristics**					
**Hours of night-time sleep**					

Normal sleep	2208	Ref.	Ref.		Ref.		Ref.	
Short sleep (≤6 h)	529	Ref.	1.05 (0.81; 1.36)		1.05 (0.77; 1.41)		-0.02 (-0.17; 0.13)	
Long sleep (≥9 h)	304	Ref.	**1.66 (1.22; 2.28)**	*	**0.62 (0.38; 1.00)**		**-0.51 (-0.71; -0.32)**	*
**Hours of day-time sleep**[‖]								
None	600	Ref.	Ref.		Ref.		Ref.	
Short nap (≤30 min)	691	Ref.	1.22 (0.90; 1.66)		1.02 (0.72; 1.44)		-0.00 (-0.17; 0.17)	
Long nap (30-60 min)	350	Ref.	1.05 (0.76; 1.46)		0.92 (0.60; 1.41)		0.04 (-0.16; 0.24)	
Very long nap (≥60min)	100	Ref.	1.05 (0.51; 2.18)		1.27 (0.66; 2.42)		0.08 (-0.24; 0.41)	
**Overall sleep quality**[§]								
Very good	430	Ref.	1.23 (0.91; 1.68)		**1.44 (1.00; 2.13)**		-0.02 (-0.18; 0.17)	
Good	1268	Ref.	Ref.		Ref.		Ref.	
Fair	429	Ref.	1.03 (0.74; 1.43)		1.01 (0.70; 1.45)		0.03 (-0.14; 0.21)	
Poor/very poor	65	Ref.	1.01 (0.49; 2.10)		0.80 (0.32; 2.02)		0.04 (-0.36; 0.44)	
**Health variables**								
**Overall health**								
1st quartile (worst)	616	Ref.	Ref.		Ref.		Ref.	
2nd quartile	556	Ref.	0.90 (0.65; 1.26)		1.01 (0.69; 1.48)		0.10 (-0.08; 0.29)	
3rd quartile	622	Ref.	0.90 (0.65; 1.25)		1.22 (0.84; 1.79)		0.16 (-0.03; 0.34)	
4th quartile (best)	1247	Ref.	0.91 (0.66; 1.25)		1.58 (1.08; 2.31)	*	0.28 (0.09; 0.46)	*
**Pain scale¶**	1323	Ref.	1.09 (0.98; 1.20)		1.01 (0.92; 1.11)		-0.01 (-0.06; 0.04)	
**Chronic morbidities**								
Diabetes	532	Ref.	0.93 (0.72; 1.21)		1.16 (0.87; 1.55)		0.08 (-0.07; 0.23)	
Hypertension	1690	Ref.	1.01 (0.82; 1.24)		**0.73 (0.58; 0.93)**		-0.08 (-0.20; 0.04)	
CVD[†]	199	Ref.	0.81 (0.54; 1.22)		0.87 (0.55; 1.39)		0.05 (-0.18; 0.29)	
Cancer[†]	188	Ref.	1.12 (0.74; 1.68)		**1.45** (0.95; 2.22)		0.11 (-0.12; 0.35)	
Osteomuscular disease	1155	Ref.	0.89 (0.71; 1.11)		0.93 (0.72; 1.19)		0.05 (-0.08; 0.18)	
Depression	368	Ref.	1.12 (0.81; 1.55)		0.92 (0.64; 1.34)		**-0.11** (-0.29; 0.08)	
**Mobility limitations**[¶]	476	Ref.	1.21 (0.78; 1.88)		0.95 (0.64; 1.43)		-0.15 (-0.37; 0.05)	
**Negative ageing experience scale**[¶]	1313	Ref.	1.11 (0.93; 1.33)		0.92 (0.78; 1.08)		-0.05 (-0.14; 0.04)	
**Cantril ladder**[‡]	1770	Ref.	1.05 (0.97; 1.15)		1.05 (0.96; 1.14)		-0.01 (-0.06; 0.04)	
**Low MMSE scores (<23)**[‡]	77	Ref.	0.68 (0.33; 1.38)		0.97 (0.43; 2.18)		-0.01 (-0.38; 0.35)	
**Years since baseline examination**	3041	Ref.	1.05 (0.86; 1.29)		1.02 (0.81; 1.28)		-0.03 (-0.15; 0.09)	

**Week of the state of alarm**	3041	Ref.	1.09 (0.99; 1.21)	1.00 (0.89; 1.13)	0.00 (-0.05; 0.07)

OR: Odds Ratio. 95%CI: 95% Confidence interval. $\bar{x}$: Mean changes. Confidence intervals are represented by the lower limit and the upper limit with either a hyphen (when the main estimates is an OR) or a semicolon (when the main estimate is a difference in means); MMSE: Mini Mental State Examination

PA: Physical activity; BMI: Body Mass Index; ST: Sedentary time

Note: Figures highlighted in bold show a statistically significant association.

†Information not available in *EXERNET*; ‡ Information only available in the *Seniors-ENRICA*-2 and *Edad con Salud* cohorts; §Information not available in the *TSHA* cohort; ǁInformation only available in the *Seniors ENRICA-2* and *Exernet* cohorts; ¶ Information only available in the *Seniors-ENRICA-2* cohort

All models were adjusted for baseline age, sex (men or women), educational level (primary or less, secondary, or university), civil status (married, widowed, never married, divorced), housing conditions (lack of outdoor views, terrace/balcony, private garden/yard, lack of internet and noise), smoking status (never, former, or current), adherence to the Mediterranean diet (MEDAS score), body mass index (normoweight, overweight, or obese), physical activity (cohort-specific quartiles), hours of sleep at night (normal, short sleep, long sleep), chronic comorbidities (hypertension, diabetes, osteo-muscular disease and depression), overall health (SF-12, WHODAS-12 o EQ5D), time since last follow-up visit, cohort of study and week of the state of alarm.

* p-value for trend <0.05

Table S4. Prospective association between participant characteristics at baseline and changes in weight between the COVID-19 pre-confinement period and the State of Alarm (n=1561: 792 from *Seniors-ENRICA*-2, 239 from *Edad con Salud*, 312 from *TSHA* and 218 from *Exernet*).

	Changes in weight				
	**n**	**Maintained**	**Increased ≥1 kilos**	**Decreased≥1 kilos**	**Mean changes (kg)**
			OR (95%CI)	OR (95%CI)	$\bar{x}$ (95%CI)
		n=480	n=465	n=616	n=1562
**Socio-demographic characteristics**					
**Age, yr**	1561	1561	1.00 (0.97; 1.03)	1.01 (0.98; 1.04)	-0.01 (-0.06; 0.03)
**Sex**					
Male	715	Ref.	Ref.	Ref.	Ref.
Female	846	Ref.	0.69 (0.46; 1.05)	0.94 (0.63; 1.39)	**-0.73 (-1.35; -0.12)**
**Education**					
Primary or less	952	Ref.	Ref.	Ref.	Ref.
Secondary	299	Ref.	1.04 (0.73; 1.50)	1.06 (0.75; 1.51)	-0.03 (-0.57; 0.51)
University	310	Ref.	0.82 (0.56; 1.19)	1.14 (0.81; 1.63)	**-0.59 (-1.14; -0.03)**
**Civil status**					
Married	1066	Ref.	Ref.	Ref.	Ref.
Never married	77	Ref.	1.04 (0.53; 2.06)	0.79 (0.40; 1.57)	0.03 (-1.01; 1.08)
Divorced	82	Ref.	1.31 (0.67; 2.58)	0.90 (0.45; 1.78)	0.53 (-0.49; 1.55)
Widowed	336	Ref.	0.85 (0.53; 1.37)	0.74 (0.47; 1.17)	0.12 (-0.60; 0.85)
**Living alone**					
No	1210	Ref.	Ref.	Ref.	Ref.
Yes	351	Ref.	1.19 (0.74; 1.91)	1.54 (0.96; 2.45)	-0.23 (-0.97; 0.48)
**Daily contact family/friends**[†]					
Yes	1089	Ref.	Ref.	Ref.	Ref.
No	251	Ref.	0.82 (0.55; 1.22)	1.07 (0.74; 1.56)	-0.17 (-0.77; 0.43)
**Feeling lonely**[‡]	1028	Ref.	1.02 (0.85; 1.23)	0.93 (0.77; 1.14)	0.13 (-0.14; 0.40)
**Housing conditions**					
**Lack outdoor views**					
No	1440	Ref.	Ref.	Ref.	Ref.
Yes	121	Ref.	1.15 (0.70; 1.89)	0.94 (0.58; 1.54)	0.26 (-0.49; 1.02)
**Lack terrace/balcony**					
No	1128	Ref.	Ref.	Ref.	Ref.
Yes	433	Ref.	0.85 (0.63; 1.15)	0.82 (0.61; 1.09)	0.24 (-0.21; 0.68)
**Lack garden/yard**					

No	392	Ref.	Ref.	Ref.	Ref.
Yes	1169	Ref.	0.83 (0.59; 1.16)	0.81 (0.59; 1.10)	0.33 (-0.16; 0.82)
**Internet access**					
Yes	1133	Ref.	Ref	Ref.	Ref.
No	428	Ref.	0.84 (0.59; 1.20)	0.99 (0.71; 1.38)	-0.29 (-0.80; 0.23)
**Too much noise**					
No	1510	Ref.	Ref.	Ref.	Ref.
Yes	51	Ref.	1.56 (0.71; 3.45)	1.61 (0.76; 3.40)	0.07 (-1.04; 1.19)
**Lifestyle-behaviours**					
**Smoking**					
Never smokers	895	Ref.	Ref.	Ref.	Ref.
Former smokers	543	Ref.	0.99 (0.72; 1.37)	0.97 (0.71; 1.31)	-0.23 (-0.71; 0.25)
Smoker	115	Ref.	**2.00 (1.18; 3.39)**	0.13 (0.65; 2.00)	0.66 (-0.15; 1.47)
**Alcohol intake**[†]					
Non drinker	614	Ref.	Ref.	Ref.	Ref.
Drinker, not daily	335	Ref.	**0.67 (0.47; 0.96)**	**0.63 (0.44; 0.90)**	0.28 (-0.29; 0.85)
Drinker, daily or almost daily	394	Ref.	0.77 (0.53; 1.11)	0.73 (0.51; 1.05)	0.22 (-0.35; 0.79)
**MEDAS**	1561	Ref.	1.05 (0.97; 1.14)	1.01 (0.93; 1.09)	0.10 (-0.02; 0.21)
**PA (cohort-specific quartiles)**					
1st quartile	369	Ref.	Ref.	Ref.	Ref.
2nd quartile	385	Ref.	1.29 (0.87; 1.92)	0.99 (0.69; 1.52)	**0.60 (0.03; 1.17)**
3rd quartile	422	Ref.	1.25 (0.85; 1.86)	0.92 (0.64; 1.32)	0.32 (-0.25; 0.90)
4th quartile	385	Ref.	1.41 (0.87; 1.96)	0.86 (0.59; 1.26)	0.49 (-0.10; 1.08)
**BMI**					
Normoweight	480	Ref.	Ref.	Ref.	Ref.
Overweight	465	Ref.	0.79 (0.52; 1.23)	1.00 (0.66; 1.53)	0.39 (-0.25; 1.04)
Obese	616	Ref.	0.92 (0.42; 1.99)	1.31 (0.64; 2.59)	0.53 (-0.57; 1.63)
**ST (cohort-specific quartiles)**[§]					
1st quartile	351	Ref.	Ref.	Ref.	Ref.
2nd quartile	293	Ref.	1.08 (0.72; 1.63)	1.26 (0.84; 1.89)	-0.07 (-0.63; 0.49)
3rd quartile	314	Ref.	1.04 (0.69; 1.56)	1.10 (0.74; 1.65)	0.05 (-0.50; 0.60)
4th quartile	291	Ref.	0.88 (0.57; 1.35)	0.93 (0.61; 1.40)	-0.10 (-0.68; 0.48)
**Sleep characteristics**					
**Hours of night-time sleep**					
Normal sleep	1168	Ref.	Ref.	Ref.	Ref.

Short sleep	259	Ref.	0.70 (0.48; 1.02)	1.04 (0.75; 1.46)		**-0.67 (-1.22; -0.13)**
Long sleep	134	Ref.	1.40 (0.83; 2.37)	1.49 (0.91; 2.43)		-0.14 (-0.87; 0.59)
**Hours of day-time sleep‖**						
None	332	Ref.	Ref.	Ref.		Ref.
Short nap	433	Ref.	0.82 (0.55; 1.23)	0.87 (0.58; 1.29)		-0.08 (-0.59; 0.43)
Long nap	185	Ref.	1.06 (0.64; 1.75)	1.06 (0.65; 1.72)		0.11 (-0.51; 0.72)
Very long nap	58	Ref.	0.65 (0.27; 1.54)	1.20 (0.56; 2.59)		**-1.29 (-2.27; -0.30)**
**Health variables**						
**Overall sleep quality§**						
Very good	234	Ref.	0.96 (0.64; 1.45)	**0.66 (0.44; 0.99)**		**0.73 (0.17; 1.29)**
Good	734	Ref.	Ref.	Ref.		Ref.
Fair	235	Ref.	0.96 (0.63; 1.44)	1.07 (0.72; 1.60)		0.16 (-0.40; 0.71)
Poor/very poor	33	Ref.	1.51 (0.51; 4.49)	1.64 (0.61; 4.42)	*	-0.10 (-1.42; 1.21)
**Overall health**						
1st quartile	267	Ref.	Ref.	Ref.		Ref.
2nd quartile	306	Ref.	0.96 (0.61; 1.51)	0.97 (0.62; 1.51)		0.04 (-0.64; 0.72)
3rd quartile	339	Ref.	0.92 (0.58; 1.43)	1.00 (0.65; 1.56)		-0.08 (-0.76; 0.60)
4th quartile (best)	649	Ref.	0.80 (0.51; 0.26)	1.01 (0.65; 1.56)		-0.18 (-0.85; 0.50)
**Pain scale ¶**	650	Ref.	1.04 (0.93; 1.17)	1.00 (0.88; 1.12)		0.09 (-0.14; 0.16)
**Chronic morbidities**						
Diabetes	256	Ref.	1.07 (0.73; 1.55)	0.93 (0.65; 1.32)		0.03 (-0.52; 0.58)
Hypertension	830	Ref.	0.98 (0.74; 1.30)	0.91 (0.69; 1.19)		0.21 (-0.21; 0.64)
CVD†	92	Ref.	1.14 (0.62; 2.13)	1.08 (0.60; 1.93)		**0.99 (0.07; 1.90)**
Cancer†	102	Ref.	1.10 (0.63; 1.91)	1.20 (0.71; 2.03)		-0.30 (-1.13; 0.52)
Osteomuscular	597	Ref.	1.**32 (0.99; 1.79)**	1.07 (0.80; 1.54)		0.36 (-0.09; 0.81)
Depression	154	Ref.	1.29 (0.80; 2.11)	1.30 (0.81; 2.08)		-0.09 (-0.79; 0.62)
**Mobility limitations¶**	259	Ref.	1.09 (0.67; 1.79)	1.06 (0.64; 1.77)		-0.07 (-0.71; 0.56)
**Negative ageing experience scale¶**	766	Ref.	1.01 (0.83; 1.23)	0.87 (0.72; 3.00)		**0.27 (0.01; 0.52)**
**Cantril ladder¶**	1025	Ref.	0.95 (0.86; 1.05)	**0.90 (0.81; 0.99)**		0.07 (-0.07; 0.21)
**Low MMSE scores (<23)¶**	26	Ref.	0.91 (0.31; 2.67)	0.80 (0.25; 2.60)		0.70 (-0.84; 2.26)
**Years since examination**	1561	Ref.	1.06 (0.78; 1.43)	1.21 (0.91; 1.60)		0.01 (-0.43; 0.45)
**Week state alarm**	1561	Ref.	0.98 (0.85; 1.13)	0.93 (0.82; 1.08)		0.05 (-0.16; 0.27)

OR: Odds Ratio. 95%CI: 95% Confidence interval; MEDAS: Mediterranean Diet Assessment Score; PA: Physical activity; ST: Sedentary time; MMSE: Mini Mental State Examination

Note: Figures highlighted in bold show a statistically significant association.

†Information not available in *EXERNET*; ‡Information only available in *Seniors ENRICA-2* and *Edad con Salud*; §Information not available in *TSHA*; ǁInformation only available in *Seniors ENRICA-2* and *Exernet* ¶Information only available in *Seniors-ENRICA-2*
All models were adjusted for baseline age, sex (men or women), educational level (primary or less, secondary, or university), civil status (married, widowed, single, divorced), smoking status (never, former, or current), alcohol drinking (never, former, moderate, or heavy), adherence to the Mediterranean diet (MEDAS score), weight and height, physical activity (quartiles), hours of sleep at night (normal, short sleep, long sleep), chronic comorbidities (hypertension, diabetes, cardiovascular disease, cancer, osteo-muscular disease and depression), overall health (SF-12, WHODAS-12 o EQ5D), time since last follow-up visit, cohort of study and week of the state of alarm.
* p-value for trend <0.05

Table S5. Prospective association between participant characteristics and changes in overall physical activity (n=2577: 1323 from *Seniors ENRICA-2*, 829 from *TSHA*, and 425 from *EXERNET*), recreational physical and household physical activity (n=1748: 1323 from *Seniors ENRICA-2* and 425 from *EXERNET*), and the PASE score (n=829 from *TSHA*) between the COVID-19 pre-confinement period and the State of Alarm.

	Changes in PA					Changes in PA in Seniors-ENRICA-2 and EXERNET		Changes in PA in TSHA
	n	Average changes	Unhealthier changes#	Healthier changes#	Total PA (Mets-h/wk)	Recreational PA (Mets-h/wk)	Household PA (Mets-h/wk)	PASE score
			OR (95%CI)	OR (95%CI)	x̄ (95%CI)	x̄ (95%CI)	x̄ (95%CI)	x̄ (95%CI)
		n=1291	n=643	n=643	n=1748	n=1748	n=1748	n=829
**Socio-demographic characteristics**								
**Age, yr**	2577	Ref.	**1.06 (1.04; 1.09)**	**0.95 (0.92; 0.97)**	**-0.61 (-0.90; -0.32)**	**-0.29 (-0.48; -0.11)**	**-0.36 (-0.57; -0.14)**	**-2.33 (-3.04; -1.62)**
**Sex**								
Male	1088	Ref.	Ref.	Ref.	Ref.	Ref.	Ref.	Ref.
Female	1489	Ref.	**0.59 (0.44; 0.78)**	**1.34 (1.03; 1.74)**	**11.1 (7.98; 14.20)**	**-4.66 (-6.54; -2.77)**	**15.2 (12.71; 17.64)**	6.32 (-2.41; 15.0)
**Education**								
Primary or less	1778	Ref.	Ref.	Ref.	Ref.	Ref.	Ref,	Ref,
Secondary	402	Ref.	1.04 (0.76; 1.44)	0.95 (0.72; 1.28)	-1.75 (-5.05;1.56)	-0.35 (-2.42; 1.72)	-1.51 (-3.97; 0.95)	5.25 (-5.48; 15.98)
University	397	Ref.	1.16 (0.81; 1.62)	1.11 (0.83; 1.49)	-0.95 (-4.40; 2.41)	-0.36 (-2.45; 2.74)	-0.73 (-3.23; 1.77)	0.02 (-13.0; 13.1)
**Civil status**								
Married	1671	Ref.	Ref.	Ref.	Ref.	Ref.	Ref.	Ref.
Never married	144	Ref.	0.85 (0.50; 1.47)	0.74 (0.44; 1.26)	-4.31 (-10.1; 1.48)	-0.89 (-4.51; 2.73)	-3.40 (-7.72; 0.92)	-1.71 (-20.2; 16.7)
Divorced	106	Ref.	0.94 (0.50; 1.78)	0.61 (0.34; 1.09)	-3.03 (-0.12; 3.07)	-0.93 (-4.74; 2.89)	-2.24 (-6.78; 2.31)	1.00 (-25.1; 27.1)
Widowed	656	Ref.	0.84 (0.57; 1.23)	0.97 (0.69; 1.35)	0.74 (-3.28; 4.76)	0.27 (-2.24; 2.78)	0.51 (-2.49; 3.51)	-2.85 (-14.4; 8.68)
**Living alone**	1944							
No	633	Ref.	Ref.	Ref.	Ref.	Ref.	Ref.	Ref.
Yes		Ref.	1.05 (0.71; 1.56)	1.23 (0.87; 1.72)	-1.22 (-5.24; 2.80)	1.20 (-1.31; 3.72)	-2.26 (-5.26; 0.75)	2.16 (-10.1; 14.4)

**Daily contact with family/friends other than cohabitants†**								
Yes	1759	Ref.	Ref.	Ref.	Ref.	Ref.	Ref.	Ref.
No	389	Ref.	1.17 (0.85; 1.62)	**0.74 (0.54;0.99)**	-1.20 (-5.17; 2.77)	-1.91 (-4.58; 0.76)	0.72 (-2.10; 3.55)	**-11.7 (-20.0; -3.53)**
**Feeling lonely**	1312	Ref.	0.97 (0.81; 1.15)	1.11 (0.95; 1.31)	0.83 (-0.68; 2.34)	-2.03 (-4.70; 0.63)	**1.31 (0.23; 2.39)**	**-**
**Housing conditions**								
**Lack of outdoor views**								
No	2391	Ref.	Ref.	Ref.	Ref.	Ref.	Ref.	Ref.
Yes	749	Ref.	**2.34 (1.56; 3.52)**	1.01 (0.69; 1.57)	**-7.17 (-11.4:-2.91)**	**-2.80 (-5.44: -0.12)**	**-4.43 (-7.60; -1.25)**	6.14 (-22.7; 10.5)
**Lack of terrace/balcony**								
No	1828	Ref.	Ref.	Ref.	. Ref	. Ref	Ref.	Ref.
Yes	749	Ref.	1.15 (0.90; 1.47)	0.90 (0.72; 1.13)	**-2.55 (-5.29; 0.00)**	**-1.03 (-2.74; 0.68)**	-1.54 (-3.59; 0.50)	-1.97 (-9.45; 5.51)
**Lack of garden/yard**								
No	778	Ref.	Ref.	Ref.	Ref.	Ref.	Ref.	Ref.
Yes	1799	Ref.	0.85 (0.64; 1.12)	**0.68 (0.54; 0.87)**	-0.60 (-3.81; 2.60)	-0.66 (-2.67; 1.35)	0.26 (-2.13; 2.66)	-6.41 (-14.0; 1.40)
**Internet access**								
Yes	1582	Ref.	Ref.	Ref.	Ref.	Ref.	Ref.	Ref.
No	995	Ref.	1.10 (0.85; 1.43)	0.90 (0.71; 1.15)	-2.29 (-5.25; 0.66)	-1.52 (-3.37; 0.33)	-0.86 (-3.07; 1.34)	0.07 (-0.72; 7.29)
**Too much noise**								
No	2506	Ref.	Ref.	Ref.	Ref.	Ref.	Ref.	Ref.
Yes	71	Ref.	1.11 (0.56; 2.22)	0.99 (0.54; 1.81)	0.13 (-7.35; 7.60)	0.52 (-5.20; 4.17)	0.33 (-5.25; 5.91)	-5.73 (-25.1; 13.6)
**Lifestyle-behaviours**								
**Smoking**								
Never smokers	1593	Ref.	Ref.	Ref.	Ref.	Ref.	Ref.	Ref.
Former smokers	181	Ref.	1.04 (0.79; 1.39)	0.83 (0.64; 1.07)	-1.70 (-4.60; 1.20)	0.73 (-1.08; 2.55)	**-2.35 (-4.52; -0.19)**	-5.58 (-15.3; 4.13)

Smoker	785	Ref.	1.06 (0.67; 1.69)	0.71 (0.46; 1.07)	-3.44 (-8.24; 1.45)	-2.81 (-5.82; 0.19)	-0.86 (-4.44; 2.70)	-11.5 (-26.1; 3.08)
**Alcohol intake**†								
Not drinker	1079	Ref.	Ref.	Ref.	Ref.	Ref.	Ref.	Ref.
Drinker, not daily	455	Ref.	0.95 (0.69; 1.30)	1.18 (0.88; 1.59)	1.40 (-1.90; 4.70)	-0.15 (-2.36; 2.07)	1.72 (-0.62; 4.07)	3.75 (-8.18; 15.68)
Drinker, daily/almost daily	618	Ref.	0.76 (0.56; 1.04)	1.01 (0.76; 1.35)	1.39 (-2.05; 4.82)	1.03 (-1.27; 3.33)	0.60 (-1.85; 3.05)	-0.36 (-9.14; 8.42)
**MEDAS**	2577	Ref.	0.96 (0.90; 103.)	**1.05** (0.99; 1.11)	**1.33 (0.66; 2.01)**	**0.73 (0.31; 1.16)**	**0.70 (0.20; 1.20)**	1.30 (-0.79; 3.39)
**BMI**								
Normoweight	561	Ref.	Ref.	Ref.	Ref.	Ref.	Ref.	Ref.
Overweight	1205	Ref.	1.06 (0.80; 1.43)	0.94 (0.72; 1.22)	0.37 (-2.56; 3.30)	1.03 (-0.81; 2.86)	-0.54 (-2.73; 1.64)	-7.45 (-18.0; 3.13)
Obese	810	Ref.	**1.37 (1.00; 1.91)**	0.96 (0.72; 1.30)	-2.89 (-6.39; 0.62)	-1.47 (-3.66; 0.72)	-1.47 (-4.08; 1.15)	-6.30 (-17.1; 4.48)
**ST (cohort-specific quartiles)‡**								
1st quartile (less time)	477	Ref.	Ref.	Ref.	Ref.	Ref.	Ref.	-
2nd quartile	417	Ref.	1.22 (0.84; 1.78)	1.06 (0.76; 1.49)	0.22 (-3.09; 3.54)	0.40 (-1.67; 2.47)	-0.13 (-2.61; 2.34)	-
3rd quartile	440	Ref.	0.97 (0.67; 1.41)	0.84 (0.60; 1.17)	-0.74 (-4.02; 2.54)	-0.44 (-1.61; 2.49)	-1.24 (-3.69; 1.21)	-
4th quartile	414	Ref.	0.94 (0.64; 1.39)	**0.67 (0.47; 0.96)**	-2.60 (-5.98; 0.77)	-1.00 (-3.11; 1.11)	-1.62 (-4.14; 0.90)	**-**
**Sleep characteristics**								
**Hours night-time sleep**								
Normal sleep	1850	Ref.	Ref.	Ref.	Ref.	Ref.	Ref.	Ref.
Short sleep (≤6 h)	474	Ref.	1.07 (0.80; 1.44)	0.87 (0.67; 1.13)	-2.82 (-5.91; 0.27)	-0.48 (-2.42; 1.45)	**-2.39 (-4.69; -0.09)**	**11.5 (2.25; 20.7)**
Long sleep (≥9 h)	253	Ref.	1.09 (0.74; 1.60)	**0.57 (0.39; 0.84)**	-4.68 (-9.99; 0.62)	-2.50 (-5.82; 0.82)	-2.25 (-6.21; 1.71)	-5.89 (-14.9; 3.20)
**Hours day-time sleep**§								
None	600	Ref.	Ref.	Ref.	Ref.	Ref.	Ref.	.
Short nap (≤30 min)	691	Ref.	0.98 (0.71; 1.36)	1.25 (0.93; 1.68)	0.88 (-1.98; 3.74)	2.32 (0.53; 4.11)	-1.38 (-3.51; 0.76)	-

Long nap (30-60 min)	350	Ref.	1.25 (0.85; 1.83)	1.01 (0.71; 1.44)	-2.57 (-5.93; 0.79)	-0.38 (-2.48; 1.72)	-2.11 (-4.62; 0.39)	-
Very long (≥60min)	100	Ref.	0.96 (0.49; 1.91)	1.04 (0.59; 1.81)	-2.77 (-8.23; 2.69)	-0.37 (-3.78; 3.04)	-2.54 (-6.61; 1.53)	-
**Overall sleep quality**‡								
Very good	236	Ref.	1.29 (0.85; 1.75)	0.86 (0.59; 1.27)	-3.19 (-6.82; 0.45)	**-2.54 (-4.81; -0.25)**	-0.51 (-3.23; 2.21)	-
Good	1048	Ref.	Ref.	Ref.	Ref.	Ref.	Ref.	-
Fair	383	Ref.	0.93 (0.65; 1.33)	1.16 (0.84; 1.59)	2.96 (-0.14; 6.07)	0.82 (-1.13; 2.77)	2.10 (-0.22; 4.42)	-
Poor/very poor	61	Ref.	0.59 (0.26; 1.36)	0.55 (0.25; 1.20)	2.75 (-4.23; 9.73)	* 1.45 (-2.92; 5.84)	* 1.02 (-4.19; 6.24)	-
**Health variables**								
**Overall health**								
1st quartile (worst)	499	Ref.	Ref.	Ref.	Ref.	Ref.	Ref.	Ref.
2nd quartile	475	Ref.	0.88 (0.60; 1.30)	1.02 (0.73; 1.43)	**6.08 (2.47; 9.69)**	1.47 (-0.79; 3.73)	**4.68 (1.98; 7.37)**	-8.06 (-25.4; 9.25)
3rd quartile	568	Ref.	1.08 (0.74; 1.57)	1.22 (0.87; 1.70)	**5.15 (1.43; 8.87)**	2.51 (0.18; 4.84)	**2.99 (0.23; 5.76)**	7.75 (-7.43; 22.92)
4th quartile (best)	1035	Ref.	0.81 (0.54; 1.20)	1.20 (0.85; 1.66)	**6.67 (2.77; 10.58)**	3.09 (0.64; 5.54)	**4.06 (1.16; 6.96)**	6.98 (-7.17; 21.1)
**Pain scale** ¶	1049	Ref.	1.00 (0.91; 1.09)	0.94 (0.86; 1.03)	-0.10 (-0.89; 0.69)	-0.33 (-0.86; 0.21)	0.20 (-0.36; 0.76)	-
**Chronic morbidities**								
Diabetes	458	Ref.	1.04 (0.78; 1.40)	**0.75 (0.57; 0.99)**	**-4.48 (-7.69; -1.27)**	**-4.33 (-6.34; -2.32)**	-0.19 (-2.58; 2.21)	-3.36 (-12.1; 5.30)
Hypertension	1504	Ref.	1.10 (0.87; 1.40)	1.15 (0.93; 1.43)	0.59 (-1.90; 3.08)	0.55 (-1.00; 2.11)	-0.06 (-1.92; 1.79)	0.30 (-7.49; 8.09)
CVD†	144	Ref.	1.05 (0.63; 1.74)	1.13 (0.73; 1.74)	0.23 (-6.39; 6.84)	-2.22 (-6.66; 2.21)	2.13 (-2.58; 6.84)	4.65 (-6.64; 15.94)
Cancer†	144	Ref.	1.23 (0.77; 1.97)	0.99 (0.63; 1.53)	-5.04 (-10.8; 0.72)	-3.68 (-7.54; 0.19)	-1.12 (-5.23; 2.98)	2.30 (-9.89; 10.62)
Osteomuscular disease	965	Ref.	0.94 (0.73; 1.21)	1.15 (0.92; 1.44)	**3.64 (0.02; 6.27)**	**2.15 (0.51; 3.79)**	1.66 (-0.29; 3.62)	-2.70 (-11.0; 5.65)

Depression	324	Ref.	1.22 (0.83; 1.79)	0.90 (0.65; 1.24)	-1.12 (-5.02; 2.79)	-1.89 (-4.44; 0.55)	0.49 (-2.42; 3.39)	**-12.6 (-24.0; -1.12)**
**Mobility limitations¶**	476	Ref.	1.27 (0.86; 1.89)	1.10 (0.76; 1.60)	-1.22 (-4.66; 2.22)	0.00 (-2.30; 2.30)	-1.93 (-4.36; 0.51)	-
**Negative ageing experience scale¶**	1313	Ref.	0.98 (0.83; 1.14)	0.99 (0.84; 1.16)	0.52 (-0.92; 1.95)	-0.16 (-0.12; 0.80)	0.62 (-0.39; 1.64)	-
**Cantril ladder¶**	1313	Ref.	1.09 (0.99; 1.20)	1.01 (0.93; 1.10)	-0.45 (-1.23;0.32)	0.12 (-0.40; 0.64)	-0.62 (-1.17; -0.07)	-
**Low MMSE scores (<23) ¶**	45	Ref.	1.10 (0.45-2.71)	0.56 (0.22-1.42)	-5.72 (-13.5;2.06)	-2.13 (-7.36; 3.10)	-3.47 (-9.03; 2.08)	**-**
**Years since baseline exam**	2577	Ref.	1.17 (0.94; 1.12)	1.10 (0.90; 1.33)	0.89 (-1.77; 3.55)	-1.89 (-4.34; 0.55)	-0.15(-2.13; 1.84)	-2.31 (-7.57; 2.95)
**Week of the state of alarm**	2577	Ref.	0.99 (0.88; 1.45)	**1.17 (1.04; 1.30)**	**1.45 (0.24; 2.66)**	**1.27 (0.51; 2.02)**	**0.19 (-0.71; 1.10)**	**8.83 (4.31; 13.4)**

OR: Odds Ratio. 95%CI: 95% Confidence interval. $\bar{x}$: Mean change. Confidence intervals are represented by the lower limit and the upper limit with either a hyphen (when the main estimates is an OR) or a semicolon (when the main estimate is a difference in means)

#Unhealthier changes were defined by decreasing physical activity more than the observed 75th percentile change. Healthier changes were defined by decreasing physical activity less than observed 25th percentile change or even increasing physical activity with confinement

MEDAS: Mediterranean Diet Assessment Score; BMI: Body Mass Index; ST: Sedentary time; MMSE: Mini Mental State Examination.

Note: Figures highlighted in bold show a statistically significant association.

†Information not available in *EXERNET*; ‡ Information not available in *TSHA*; §Information only available in *Seniors ENRICA-2* and *Exernet*; ¶Information only available in *Seniors-ENRICA-2*

All models were adjusted for baseline age, sex (men or women), educational level (primary or less, secondary, or university), civil status (married, widowed, never married, divorced), smoking status (never, former, or current), alcohol drinking (never, former, moderate, or heavy), adherence to the Mediterranean diet (MEDAS score), body mass index (normoweight, overweight, or obese), physical activity (quartiles), hours of sleep at night (normal, short sleep, long sleep), chronic comorbidities (hypertension, diabetes, cardiovascular disease, cancer, osteo-muscular disease and depression), overall health (SF-12, WHODAS-12 or EQ5D), time since last follow-up visit, cohort of study and week of the state of alarm.

* p-value for trend <0.05

Table S6. Prospective association between participant characteristics at baseline and changes in overall sedentary time (n=1746: 1321 from Seniors-ENRICA-2 and 425 from Exernet), as well as in specific sedentary activities (n=1270 for TV viewing, 1196 for other screen time and 1297 for reading time in participants from the Seniors-ENRICA-2) between the COVID-19 pre-confinement period and the State of Alarm.

	Changes in sedentary time					Changes in sedentary time (hours /day) in Seniors-ENRICA-2		
	**n**	**Average changes**	**Unhealthier changes#**	**Healthier changes#**	**Mean changes overall sedentary time (hours/day)**	**Mean changes TV viewing time**	**Mean changes other screen time**	**Mean changes reading time**
			OR (95%CI)	OR (95%CI)	$\bar{x}$ (95%CI)	$\bar{x}$ (95%CI)	$\bar{x}$ (95%CI)	$\bar{x}$ (95%CI)
		n=880	n=431	n=437	n=1748	n=1270	n=1196	n=1297
**Socio-demographic characteristics**								
**Age, yr**	1746	Ref.	**0.96 (0.93;0.99)**	**1.04 (1.01;1.08)**	**-0.07 (-0.11; -0.04)**	**-0.03 (-0.06; -0.00)**	**-0.05 (-0.07; -0.02)**	0.01 (-0.00; 0.02)
**Sex**								
Male	757	Ref.	Ref.	Ref.	Ref.	Ref.	Ref.	Ref.
Female	989	Ref.	1.09 (0.79;1.51)	**1.54 (1.08;2.19)**	**-0.47 (-0.85; -0.10)**	**-0.31 (-0.60; -0.02)**	0.08 (-0.14; 0.30)	**0.19 (0.03; 0.45)**
**Education**								
Primary or less	1118	Ref.	Ref.	Ref.	Ref.	**Ref.**	Ref.	Ref.
Secondary	299	Ref.	1.14 (0.81;1.61)	0.98 (0.68;1.40)	0.03 (-0.36; 0.43)	-0.28 (-0.58; 0.02)	0.14 (-0.08; 0.36)	-0.06 (-0.58; 0.02)
University	329	Ref.	1.13 (0.80;1.60)	0.72 (0.49;1.05)	**0.44 (0.04; 0.85)**	**-0.50 (-0.78; -0.19)**	**0.36 (0.15; 0.57)**	0.08 (-0.08; 0.26)
**Civil status**								
Married	1107	Ref.	Ref.	Ref.	Ref.	Ref,	Ref.	Ref.
Never married	109	Ref.	0.81 (0.43;1.52)	0.95 (0.50;1.79)	-0.06 (-0.75; 0.64)	0.15 (-0.37; 0.67)	0.23 (-0.18; 0.63)	-0.14 (-0.43; 0.15)
Divorced	89	Ref.	1.32 (0.73;2.40)	0.67 (0.33;1.38)	0.63 (-0.10; 1.37)	**0.84 (0.30; 1.37)**	0.07 (-0.33; 0.46)	-0.07 (-0.37; 0.23)
Widowed	441	Ref.	0.90 (0.58;1.39)	0.81 (0.52;1.25)	0.08 (-0.40; 0.57)	0.19 (-0.20; 0.57)	0.13 (-0.16; 0.42)	-0.02 (-0.23; 0.19)
**Living alone**								
No	1289	Ref.	Ref.	Ref.	Ref.	Ref.	Ref.	Ref.
Yes	457	Ref.	1.09 (0.71;1.67)	0.88 (0.57;1.37)	0.26 (-0.23; 0.74)	-0.21 (-0.60; 0.18)	0.03 (-0.27; 0.33)	0.06 (-0.15; 0.27)

**Daily contact** †								
Yes	1147	Ref.	Ref.	Ref.	Ref.	Ref.	Ref.	Ref.
No	171	Ref.	1.04 (0.66;1.64)	0.89 (0.58;1.37)	0.10 (-0.38; 0.58)	-0.14 (-0.47; 0.19)	0.05 (-0.20; 0.29)	-0.02 (-0.21; 0.16)
**Feeling lonely** ‡	**1312**	Ref.	0.89 (0.75;1.06)	0.92 (0.78;1.09)	-0.05 (-0.23; 0.13)	-0.02 (-0.15; 0.10)	0.00 (-0.09; 0.10)	-0.03 (-0.10; 0.04)
**Housing conditions**								
**Lack of outdoor views**								
No	1596	Ref.	Ref.	Ref.	Ref.	Ref.	Ref.	Ref.
Yes	150	Ref.	1.07 (0.70;1.63)	0.88 (0.44;1.44)	0.05 (-0.46; 0.56)	**0.88 (0.15; 1.59)**	0.18 (-0.12; 0.48)	-0.05 (-0.28; 0.17)
**Lack terrace/balcony**								
No	1305	Ref.	Ref.	Ref.	Ref.	Ref.	Ref.	Ref.
Yes	441	Ref.	0.85 (0.64;1.14)	1.09 (0.82;1.47)	-0.20 (-0.53; 1.13)	-0.05 (-0.30; 0.20)	0.01 (-0.10; 0.29)	-0.09 (-0.23; 0.05)
**Lack garden/yard**								
No	295	Ref.	Ref.	Ref.	Ref.	Ref.	Ref.	Ref.
Yes	1451	Ref.	1.29 (0.92;1.80)	).81(0.58;1.17)	**0.37** (-0.01; 0.76)	**0.35 (0.03; 0.68)**	**0.18 (-0.00; 0.43)**	0.08 (-0.10; 0.26)
**Internet access**								
Yes	1206	Ref.	Ref.	Ref.	Ref.	Ref.	Ref.	Ref.
No	540	Ref.	0.89 (0.66;1.22)	0.71 (0.29;1.81)	**-0.50 (-0.85; -0.13)**	0.28 (-0.01; 0.58)	**-0.57 (-0.82; -0.32)**	-0.01 (0.45; 0.36)
**Too much noise**								
No	1701	Ref.	Ref.	Ref.	Ref.	Ref.	Ref.	Ref.
Yes	45	Ref.	1.61 (0.81 3.22)	**1.50 (1.08;2.07)**	0.77 (-0.13; 1.67)	0.02 (-0.38; 0.42)	0.23 (-0.29; 0.74)	-0.04 (0.45; 0.37)
**Lifestyle-behaviours**								
**Smoking**								
Never smokers	1013	Ref.	Ref.	Ref.	Ref.	Ref.	Ref.	Ref.
Former smokers	591	Ref.	1.04 (0.77;1.40)	0.99 (0.72;1.37)	0.10 (-0.25; 0.45)	0.09 (-0.32; 0.49)	0.05 (-0.14; 0.24)	0.07 (-0.07; 0.21)

Smoker	125	Ref.	1.31 (0.80;2.15)		0.73 (0.43;1.26)	0.43 (-0.15; 1.01)		-0.04 (-0.29; 0.22)	0.09 (-0.02; 0.28)	0.11 (-0.11; 0.33)
**Alcohol intake†**										
Non drinker	562	Ref.	Ref.		Ref.	Ref.		Ref.	Ref.	Ref.
Drinker, not daily	370	Ref.	0.94 (0.65;1.36)		1.06 (0.74;1.52)	-0.08 (-0.48; 0.32)		-0.07 (-0.35; 0.20)	0.04 (-0.17; 0.24)	0.12 (-0.04; 0.26)
Drinker, daily/almost daily	389	Ref.	0.93 (0.64;1.37)		0.83 (0.57;1.22)	0.06 (-0.35; 0.48)		0.00 (-0.29; 0.29)	-0.06 (-0.27; 0.15)	0.03 (-0.13; 0.18)
**MEDAS**	1746	Ref.	**0.93 (0.87;0.99)**		0.94 (0.87;1.01)	-0.03 (-0.11; 0.06)		-0.03 (-0.10; 0.03)	0.00 (-0.04; 0.05)	0.00 (-0.03; 0.05)
**PA (cohort-specific quartiles)**										
1st quartile	462	Ref.	Ref.		Ref.	Ref.		**Ref.**	Ref.	Ref.
2nd quartile	444	Ref.	1.00 (0.72;1.41)		1.22 (0.84;1.78)	-0.30 (-0.70; 0.10)		-0.29 (-0.60; 0.02)	-0.03 (-0.26; 0.20)	0.12 (-0.05; 0.30)
3rd quartile	457	Ref.	0.80 (0.57;1.14)		1.30 (0.89;1.91)	-0.42 (-0.83; 0.01)		**-0.33 (-0.65; -0.00)**	-0.01 (-0.25; 0.23)	-0.01 (-0.19; 0.17)
4th quartile	383	Ref.	0.71 (0.48;1.04)	*	1.17 (0.78;1.77)	-0.55 (-0.97; 0.09)	*	-0.32 (-0.66; 0.03)	-0.20 (-0.46; 0.06)	-0.04 (-0.23; 0.15)
**BMI**										
Normoweight	444	Ref.	Ref.		Ref.	Ref.		Ref.	Ref.	Ref.
Overweight	850	Ref.	1.33 (0.98;1.81)		0.93 (0.68;1.29)	0.19 (-0.16; 0.54)		0.08 (-0.18; 0.35)	0.13 (-0.07; 0.32)	-0.08 (-0.23; 0.06)
Obese	452	Ref.	1.32 (0.91;1.92)		0.99 (0.67;1.45)	0.15 (-0.27; 0.58)		0.30 (-0.03; 0.63)	**0.26 (0.02; 0.51)**	-0.16 (-0.34; 0.00)
**Sleep characteristics**										
**Hours night-time sleep‡**										
Normal sleep	1329	Ref.	Ref.		Ref.	Ref.		Ref.	Ref.	Ref.
Short sleep (≤6 h)	324	Ref.	1.03 (0.75;1.42)		0.90 (0.64;1.26)	0.16 (-0.21; 0.54)		-0.06 (-0.36; 0.24)	-0.04 (-0.26; 0.18)	**-0.17 (-0.37; 0.00)**
Long sleep (≥9 h)	93	Ref.	1.02 (0.60;1.74)		0.71 (0.38;1.34)	0.33 (-0.31; 0.96)		-0.02 (-0.55; 0.52)	-0.08 (-0.50; 0.34)	0.22 (-0.07; 0.51)
**Hours day-time sleep**										

None	599	Ref.	Ref.	Ref.		Ref.		Ref.	Ref.	Ref.
Short nap (≤30 min)	691	Ref.	1.53 (1.13;2.07)	0.85 (0.62;1.16)		**0.55 (0.22; 0.90)**		0.03 (-0.23; 0.29)	0.07 (-0.09; 0.23)	0.07 (-0.09; 0.23)
Long nap (30-60 min)	349	Ref.	1.15 (0.81;1.66)	0.72 (0.50;1.05)		**0.58 (0.17; 0.98)**		0.17 (-0.16; 0.50)	0.11 (-0.10; 0.32)	0.11 (-0.10; 0.32)
Very long nap (≥60min)	100	Ref.	1.58 (0.89;2.82)	**0.52 (0.28;0.96)**	*	**1.06 (0.41; 1.72)**	*	0.40 (-0.12; 0.92)	0.08 (-0.25; 0.40)	0.08 (-0.25; 0.40)
**Overall sleep quality**										
Very good	235	Ref.	1.13 (0.79;1.63)	0.98 (0.65;1.48)		-0.04 (-0.47; 0.40)		0.07 (-10.9; 0.33)	0.05 (-0.32; 0.41)	-0.08 (-0.31; 0.14)
Good	1047	Ref.	Ref.	Ref.		Ref.		Ref.	Ref.	Ref.
Fair	383	Ref.	0.70 (0.50;0.99)	0.95 (0.68;1.33)		-0.30 (-0.67; 0.07)		0.04 (-0.16; 0.25)	0.13 (-0.16; 0.42)	-0.21(-0.38; -0.03)
Poor/very poor	61	Ref.	1.12 (0.56;2.23)	0.51 (0.22;1.19)		0.26 (-0.58; 1.10)		0.04 (-0.52; 0.59)	-0.11 (-0.90; 0.68)	-0.21 (-0.70; 0.27)
**Health variables**										
**Overall health**										
1st quartile (worst)	422	Ref.	Ref.	Ref.		Ref.		Ref.	Ref.	Ref.
2nd quartile	415	Ref.	1.39 (0.94;2.06)	1.05 (0.72;1.54)		0.13 (-0.30; 0.57)		-0.09 (-0.43; 0.25)	-0.11 (-0.36; 0.14)	0.19 (0.01; 0.38)
3rd quartile	444	Ref.	1.23 (0.83;1.84)	0.88 (0.59;1.31)		0.28 (-0.17; 0.72)		-0.12 (-0.47; 0.23)	0.00 (-0.25; 0.27)	**0.17 (-0.00; 0.36)**
4th quartile (best)	465	Ref.	1.17 (0.77;1.77)	0.74 (0.49;1.13)		0.26 (-0.21; 0.73)		-0.20 (-0.58; 0.18)	0.14 (-0.14; 0.41)	**0.27 (-0.07; 0.48)**
**Pain scale‡**	1047	Ref.	1.04 (0.95;1.14)	0.96 (0.88;1.04)		0.03 (-0.07; 0.13)		**0.09 (0.02; 0.15)**	-0.01 (-0.06; 0.04)	-0.02 (-0.05; 0.02)
**Chronic morbidities**										
Diabetes	300	Ref.	1.06 (0.76;1.49)	**0.70 (0.49;0.99)**		0.30 (-0.08; 0.69)		**0.40 (0.11; 0.70)**	**0.23 (0.00; 0.46)**	**-0.21 (-0.37; -0.04)**
Hypertension	922	Ref.	1.09 (0.84;1.41)	1.01 (0.77;1.33)		0.09 (-0.20; 0.39)		-0.04 (-0.27; 0.19)	-0.15 (-0.32; 0.02)	0.09 (-0.03; 0.22)
CVD†	56	Ref.	1.67 (0.81;3.48)	1.07 (0.53;2.16)		0.55 (-0.25; 1.35)		0.02 (-0.53; 0.57)	0.01 (-0.41; 0.44)	-0.00 (-0.31; 0.30)
Cancer†	72	Ref.	0.86 (0.42;1.74)	1.50 (0.82;2.74)		-0.62 (-1.32; 0.08)		0.06 (-0.44; 0.55)	**-0.43 (-0.78; -0.08)**	0.01 (-0.25; 0.38)

Osteomuscular disease	753	Ref.	**0.75 (0.57;0.99)**	1.12 (0.84;1.49)	-0.30 (-0.62; 0.01)	0.01 (-0.18; 0.35)	-0.06 (-0.24; 0.12)	-0.17 (-0.38; 0.04)
Depression	220	Ref.	0.96 (0.62;1.48)	1.41 (0.94;2.12)	-0.38 (-0.85; 0.09)	-0.23 (-0.61; 0.16)	-0.00 (-0.30; 0.29)	-0.04 (-0.18; 0.11)
**Mobility limitations‡**	475	Ref.	0.72 (0.48;1.06)	0.75 (0.51;1.09)	0.09 (-0.33; 0.51)	**0.38 (0.10; 0.67)**	0.08 (-0.14; 0.29)	-0.00 (-0.16; 0.16)
**Negative ageing experience scale‡**	1311	Ref.	0.91 (0.77;1.07)	0.97 (0.83;1.14)	-0.08 (-0.25; 0.10)	0.00 (-0.11; 0.13)	-0.05 (-0.14; 0.04)	-0.02 (-0.09; 0.04)
**Cantril ladder‡**	1311	Ref.	0.98 (0.90;1.07)	1.04 (0.95;1.13)	-0.05 (-0.14; 0.05)	-0.04 (-01.0; 0.03)	0.02 (-0.03; 0.07)	-0.01 (-0.05; 0.03)
**Low MMSE (<23) ‡**	45	Ref.	0.42 (0.13;1.33)	0.79 (0.35;1.81)	-0.50 (-1.45; 0.44)	-0.10 (-0.76; 0.54)	-0.41 (-1.03; 0.20)	-0.16 (-0.53; 0.20)
**Years since examination**	1746	Ref.	0.96 (0.73;1.26)	0.78 (0.58;1.05)	-0.07 (-0.39; 0.25)	-0.01 (-0.28; 0.25)	-0.02 (0.21; 0.18)	-0.02 (-0.08; 0.04)
**Week of state alarm**	1746	Ref.	**0.89 (0.78;1.00)**	1.12 (0.99;1.27)	**-0.30 (-0.44; -0.15)**	**-0.12 (-0.23; -0.00)**	**-0.10 (-0.19; -0.02)**	**-0.21(-0.37; -0.04)**

OR: Odds Ratio. 95%CI: 95% Confidence interval. $\bar{x}$: Mean change. Confidence intervals are represented by the lower limit and the upper limit with either a hyphen (when the main estimates is an OR) or a semicolon (when the main estimate is a difference in means)

MEDAS: Mediterranean Diet Assessment Score; BMI: Body Mass Index; PA: Physical activity; MMSE: Mini Mental State Examination.

Note: Figures highlighted in bold show a statistically significant association.

#Unhealthier changes were defined by increasing sedentary time more than the observed 75th percentile change. Healthier changes were defined by increasing sedentary time less than observed 25th percentile change

‡Information not available in *EXERNET*; ‡Information only available in *Seniors ENRICA-2*; §. Information only available in *Seniors ENRICA*-2 and *Edad con Salud* cohorts.

All models were adjusted for baseline age, sex (men or women), educational level (primary or less, secondary, or university), civil status (married, widowed, never married, divorced), smoking status (never, former, or current), alcohol drinking (never, former, moderate, or heavy), adherence to the Mediterranean diet (MEDAS score), body mass index (normoweight, overweight, or obese), physical activity (quartiles), hours of sleep at night (normal, short sleep, long sleep), chronic comorbidities (hypertension, diabetes, cardiovascular disease, cancer, osteo-muscular disease and depression), overall health (SF-12, WHODAS-12 o EQ5D),time since last follow-up visit, cohort of study and week of the state of alarm.

*p-value for trend <0.05

Table S7 Prospective association between participant characteristics and changes in night-time sleep (n=2867, 1294 from *Seniors-ENRICA-2*, 411 from *Edad con Salud*, 763 from *TSHA* and 399 from *Exernet*), sleep quality (n=2095; 1289 from *Seniors-ENRICA-2*, 430 from *Edad con Salud*, and 376 from *Exernet*), and nº of poor sleep quality indicators (n=1285 in *Seniors-ENRICA 2*) during the COVID-19 confinement.

	**Changes in night-time sleep**				**Changes in sleep quality**					
	**n**	**No changes**	**Worsening** [#]	**Improvement** [#]	**n**	**No changes**	**Worsening** [##]	**Improvements** [##]	**n**	**Changes in nº of poor sleep indicators**[###]
			OR (95%CI)	OR (95%CI)			OR (95%CI)	OR (95%CI)		$\bar{x}$ (95%CI)
		n=971	n=2020	n=802		n=1180	n=282	n=633		n=1285
**Socio-demographic characteristics**										
**Age, yr**	2867	Ref.	1.00 (0.98; 1.02)	1.00 (0.98; 1.02)	2095	Ref.	0.99 (0.96; 1.02)	1.01 (0.98; 1.03)	1285	0.00 (-0.01; 0.02)
**Sex**										
Male	1225	Ref.	Ref.	Ref.	906	Ref.	Ref.	Ref	645	Ref.
Female	1642	Ref.	0.93 (0.74; 1.18)	1.06 (0.82; 1.37)	1189	Ref.	1.26 (0.88; 1.79)	0.85 (0.66; 1.10)	640	0.11 (-0.05; 0.27)
**Education**										
Primary or less	1894	Ref.	Ref.	Ref.	1289	Ref.	Ref.	Ref.	746	Ref.
Secondary	511	Ref.	0.89 (0.68; 1.17)	1.15 (0.87; 1.53)	413	Ref.	0.79 (0.54; 1.15)	0.96 (0.73; 1.25)	249	0.00 (-0.16; 0.17)
University	462	Ref.	0.81 (0.60; 1.09)	1.10 (0.80; 1.50)	393	Ref.	0.78 (0.51; 1.18)	0.93 (0.70; 1.24)	290	-0.06 (-0.23; 0.11)
**Civil status**										
Married	1876	Ref.	Ref;	Ref.	1340	Ref.	Ref.	Ref.	839	Ref.
Never married	155	Ref.	0.94 (0.57; 1.56)	1.02 (0.61; 1.71)	124	Ref.	0.49 (0.22; 1.09)	**1.64 (1.00; 2.67)**	88	0.05 (-0.26; 0.35)
Divorced	149	Ref.	1.01 (0.61; 1.67)	1.20 (0.72; 1.99)	134	Ref.	0.99 (0.53; 1.85)	1.43 (0.89; 2.29)	77	0.02 (-0.29; 0.33)
Widowed	687	Ref.	1.13 (0.81; 1.56)	1.02 (0.72; 1.44)	497	Ref.	0.83 (0.52; 1.34)	1.32 (0.93; 1.88)	281	0.11 (-0.11; 0.33)
**Living alone**										
No	2188	Ref.	Ref.	Ref.	1562	Ref.	Ref.	Ref.	977	Ref.
Yes	679	Ref.	0.94 (0.67; 1.31)	1.11 (0.79; 1.58)	533	Ref.	1.49 (0.93; 2.38)	0.78 (0.56; 1.11)	308	0.01 (-0.22; 0.23)
**Daily contact family/friends**[†]										
Yes	1911	Ref.	Ref.	Ref.	1350	Ref.	Ref.	Ref.	1122	Ref.

No	553	Ref.	1.06 (0.82; 1.36)	0.80 (0.60; 1.08)	366	Ref.	**1.33 (1.00; 2.14)**	**1.35 (1.00; 1.81)**	160	-0.07 (-0.26; 0.13)
**Feeling lonely (1-5 scale)[‡]**	1694	Ref.	1.10 (0.97; 1.26)	0.95 (0.81; 1.12)	1717	Ref.	1.09 (0.94; 1.26)	**0.86 (0.75; 0.98)**		**0.11 (0.04; 0.18)**
**Housing conditions**										
**Lack of outdoor views**										
No	2655	Ref.	Ref.	Ref.	1914	Ref.	Ref	Ref.	1182	Ref.
Yes	212	Ref.	1.16 (0.81; 1.67)	1.15 (0.77; 1.70)	181	Ref.	**1.50 (1.00; 2.34)**	1.18 (0.82; 1.71)	103	-0.10 (-0.34; 0.13)
**Lack of terrace/balcony**										
No	2020	Ref.	Ref.	Ref.	1535	Ref.	Ref.	Ref.	959	Ref.
Yes	847	Ref.	1.10 (0.89; 1.36)	1.22 (0.97; 1.52)	560	Ref.	1.10 (0.81; 1.49)	1.09 (0.87; 1.38)	326	0.01 (-0.14; 0.15)
**Lack of garden/yard**										
No	834	Ref.	Ref.	Ref.	398	Ref.	Ref.	Ref.	173	Ref.
Yes	2033	Ref.	0.59 (0.47; 0.74)	1.86 (0.67; 1.09)	1697	Ref.	0.86 (0.61; 1.25)	0.82 (0.64; 1.07)	1112	-0.10 (-0.34; 0.13)
**Internet access**										
Yes	1865	Ref.	Ref.	Ref.	1499	Ref.	Ref.	Ref.	1026	Ref.
No	1002	Ref.	1.37 (1.09; 1.72)	1.22 (0.96; 1.56)	596	Ref.	1.10 (0.79; 1.54)	0.86 (0.66; 1.12)	259	0.05 (-0.11; 0.22)
**Too much noise**										
No	2778	Ref.	Ref.	Ref.	2034	Ref.	Ref.	Ref.	1254	Ref.
Yes	89	Ref.	1.03 (0.60; 1.76)	0.75 (0.41; 1.40)	61	Ref.	**1.99 (1.00; 4.07)**	1.56 (0.86; 2.85)	31	**0.47 (0.05; 0.89)**
**Lifestyle-behaviours**										
**Smoking**										
Never smokers	1726	Ref.	Ref.	Ref.	1193	Ref.	Ref.	Ref.	660	Ref.
Former smokers	242	Ref.	0.95 (0.75; 1.22)	1.03 (0.79; 1.35)	700	Ref.	0.97 (0.69; 1.36)	1.18 (0.92; 1.51)	509	0.02 (-0.13; 0.16)
Smoker	881	Ref.	0.95 (0.65; 1.39)	**1.73 (1.19; 2.50)**	193	Ref.	0.77 (0.45; 1.33)	1.07 (0.73; 1.55)	116	-0.12 (-0.35; 0.11)
**Alcohol intake**										
Non drinker	1231	Ref	Ref.	Ref.	778	Ref.	Ref.	Ref.	542	Ref.
Drinker, not daily	566	Ref	0.83 (0.63; 1.10)	1.02 (0.75; 1.38)	485	Ref.	1.16 (0.79; 1.70)	**1.47 (1.10; 1.94)**	360	0.07 (-0.08; 0.23)
Drinker, daily	671	Ref	0.82 (0.63; 1.08)	0.97 (0.72; 1.31)	456	Ref.	1.46 (0.97; 2.21)	1.17 (0.86.1.59)	383	0.14 (-0.03; 0.30)

**MEDAS**		Ref	1.03 (0.97; 1.09)	0.98 (0.92; 1.04)	2095	Ref.	0.94 (0.87; 1.02)		**1.10 (1.03; 1.16)**	1285	0.00 (-0.15; 0.15)
**PA (cohort-specific quartiles)**											
1st quartile (less)	725	Ref.	Ref.	Ref.	531	Ref.	Ref.		Ref.	334	Ref.
2nd quartile	740	Ref.	0.77 (0.59; 1.02)	0.89 (0.67; 1.18)	541	Ref.	1.24 (0.83; 1.86)		0.85 (0.65; 1.13)	331	0.11 (-0.06; 0.29)
3rd quartile	743	Ref.	0.77 (0.58; 1.01)	0.81 (0.60; 1.09)	550	Ref.	**1.62 (1.09; 2.42)**		0.86 (0.64; 1.15)	332	**0.22 (0.03; 0.41)**
4th quartile	659	Ref.	0.86 (0.64; 1.14)	0.90 (0.66; 1.22)	473	Ref.	1.45 (0.94; 2.21)	*	0.82 (0.61; 1.11)	288	**0.30 (0.10; 0.49)**
**BMI**											
Normoweight	649	Ref	Ref.	Ref.	551	Ref.	Ref.		Ref.	356	Ref.
Overweight	1341	Ref	0.95 (0.74; 1.22)	1.13 (0.86; 1.49)	992	Ref.	0.84 (0.60; 1.19)		0.91 (0.71; 1.16)	635	0.05 (-0.11; 0.20)
Obese	877	Ref	0.86 (0.65; 1.13)	1.12 (0.83; 1.52)	552	Ref.	0.84 (0.57; 1.23)		**0.74 (0.55; 1.00)**	294	0.07 (-0.12; 0.26)
**ST (cohort-specific quartiles)**§											
1st quartile	610	Ref.	Ref.	Ref.	613	Ref.	Ref.		Ref.	338	Ref.
2nd quartile	490	Ref.	0.80 (0.58; 1.10)	0.93 (0.65; 1.32)	480	Ref.	1.25 (0.86; 1.80)		1.13 (0.85; 1.51)	307	0.08 (-0.10; 0.26)
3rd quartile	528	Ref.	0.77 (0.56; 1.06)	0.92 (0.66; 1.29)	523	Ref.	0.94 (0.64; 1.38)		**1.41 (1.07; 1.86)**	325	0.03 (-0.15; 0.21)
4th quartile	476	Ref.	0.95 (0.69; 1.30)	0.86 (0.60; 1.24)	479	Ref.	1.04 (0.71; 1.54)		1.08 (0.81; 1.45)	315	0.02 (-0.17; 0.20)
**Sleep characteristics**											
**Night-time sleep**											
Normal sleep	2140	-	;	;	1614	Ref.	Ref.		Ref.	1024	Ref.
Short sleep (≤6 h)	488	-	;	;	351	Ref.	**1.51 (1.08; 2.13)**		1.30 (0.98; 1.71)	206	**0.39 (0.20, 0.58)**
Long sleep (≥9 h)	239	-	;	;	130	Ref.	0.70 (0.37; 1.35)		0.99 (0.65; 1.49)	55	-0.12 (-0.44; 0.19)
**Day-time sleep**‖											
No	582	Ref.	Ref.	Ref.	573	Ref.	Ref.		Ref.	388	Ref.
Short nap	673	Ref.	0.78 (0.56; 1.08)	1.04 (0.74; 1.48)	669	Ref.	0.98 (0.68; 1.40)		0.83 (0.63; 1.10)	610	0.07 (-0.07; 0.22)
Long nap	339	Ref.	1.12 (0.78; 1.61)	1.06 (0.72; 1.57)	89	Ref.	0.92 (0.60; 1.43)		0.78 (0.57; 1.11)	217	0.05 (-0.14; 0.26)

Very long nap	94	Ref.	1.18 (0.64; 2.17)		1.71 (0.94; 3.12)	27	Ref.	0.86 (0.39; 1.88)	0.77 (0.46; 1.31)	67	-0.04 (-0.35; 0.27)
**Overall sleep quality§**											
Very good	408	Ref.	0.99 (0.73; 1.33)		0.64 (0.44; 0.93)	411	Ref.	**;**	**;**	140	**-**
Good	1210	Ref.	Ref.		Ref.	1212	Ref.	**;**	**;**	834	**-**
Fair	409	Ref.	**0.52 (0.37; 0.74)**		**1.52 (1.12; 2.07)**	411	Ref.	;	;	283	-
Poor/very poor	57	Ref.	**0.18 (0.05; 0.60)**	*	0.88 (0.42; 1.85)	61	Ref.	;	;	28	-
**Overall health**											
1st quartile	544	Ref.	Ref.		Ref.	594	Ref.	Ref.	Ref.		Ref.
2nd quartile	544	Ref.	0.85 (0.61; 1.17)		0.88 (0.63; 1.25)	453	Ref.	0.86 (0.59; 1.27)	0.91 (0.66; 1.25)	309	**-0.28 (-0.47; -0.08)**
3rd quartile	597	Ref.	0.89 (0.65; 1.23)		0.89 (0.63; 1.26)	503	Ref.	0.73 (0.48; 1.11)	0.80 (0.57; 1.11)	329	**-0.44 (-0.64; -0.23)**
4th quartile (best)	1182	Ref.	**0.73 (0.53; 1.00)**	*	0.82 (0.58; 1.16)	545	Ref.	0.70 (0.46; 1.08)	1.15 (0.83; 1.59)	340	**-0.40 (-0.62; -0.18)**
**Pain scale¶**	1025	Ref.	1.01 (0.92; 1.11)		1.01 (0.92; 1.12)	1029	Ref.	**1.11 (1.01; 1.23)**	1.05 (0.97; 1.15)	307	**0.06 (0.03; 0.10)**
**Chronic morbidities**											
Diabetes	490	Ref.	0.92 (0.71; 1.20)		1.09 (0.83; 1.43)	346	Ref.	1.33 (0.94; 1.89)	0.97 (0.72; 1.29)	230	0.07 (-0.10; 0.24)
Hypertension	1582	Ref.	1.00 (0.82; 1.23)		1.12 (0.90; 1.39)	1046	Ref.	**1.47 (1.10; 1.96)**	1.11 (0.90; 1.38)	699	**0.15 (0.02-0.28)**
CVD†	178	Ref.	0.67 (0.44; 1.02)		0.76 (0.49; 1.18)	102	Ref.	1.18 (0.64; 2.17)	0.93 (0.56; 1.53)	53	-0.03 (-0.35; 0.29)
Cancer†	173	Ref.	1.27 (0.86; 1.88)		1.20 (0.78; 1.83)	112	Ref.	1.18 (0.66; 2.14)	1.30 (0.83; 2.04)	71	-0.05 (-0.33; 0.23)
Osteomuscular disease	1089	Ref.	0.89 (0.71; 1.10)		0.97 (0.77; 1.23)	895	Ref.	0.87 (0.65; 1.17)	0.87 (0.69; 1.09)	620	**0.18 (0.04; 0.32)**
Depression	342	Ref.	0.99 (0.72; 1.36)		1.05 (0.75; 1.46)	243	Ref.	**1.58 (1.05; 2.39)**	**1.52 (1.07; 2.15)**	155	**0.25 (0.03; 0.47)**
**Mobility limitations¶**	459	Ref.	0.65 (0.63; 0.98)		1.15 (0.74; 1.77)	458	Ref.	1.19 (0.96; 1.85)	1.21 (0.84; 1.74)	458	-0.08 (-0.25; 0.08)
**Negative ageing experience scale**	1284	Ref.	0.99 (0.84; 1.16)		1.05 (0.88; 1.27)	1287	Ref.	1.11 (0.93; 1.34)	0.93 (0.80; 1.08)	1287	0.05 (-0.02; 0.12)
**Cantril ladder‡**	1691	Ref.	1.03 (0.96; 1.11)		1.01 (0.93; 1.10)	1710	Ref.	0.99 (0.91; 1.08)	**1.11 (1.03; 1.19)**	1287	-0.03 (-0.06; 0.01)

**Low MMSE scores (<23)**‡	63	Ref.	0.90 (0.47; 1.73)	0.79 (0.33; 1.88)	56	Ref.	1.04 (0.45; 2.41)	1.27 (0.66; 2.42)	34	0.35 (-0.06; 0.75)
**Years since baseline examination**	2867	Ref.	0.86 (0.70; 1.05)	1.04 (0.93; 1.15)	2095	Ref.	0.81 (0.58; 1.13)	0.97 (0.87; 1.07)	1285	0.02 (-0.05; 0.08)
**Week state of alarm**	2867	Ref.	0.97 (0.86; 1.08)	0.88 (0.72; 1.09)	2095	Ref.	0.99 (0.86; 1.13)	0.84 (0.65; 1.09)	1285	0.00 (-0.15; 1.92)

OR: Odds Ratio; 95%CI: 95% Confidence interval; $\bar{x}$: Mean change. Confidence intervals are represented by the lower limit and the upper limit with either a hyphen (when the main estimates is an OR) or a semicolon (when the main estimate is a difference in means)

MEDAS: Mediterranean Diet Assessment Score; BMI: Body Mass Index; PA: Physical activity; ST: Sedentary time; MMSE: Mini Mental State Examination.

Note: Figures highlighted in bold show a statistically significant association.

Note: Figures highlighted in bold show a statistically significant association.

#Worsening was defined by having a healthy night-time time sleep duration (6-9 hours) in the pre-confinement period but not during confinement. Improvements were defined by sleeping less than 6 hours or more than 9 in the pre-confinement period and 6-9 during confinement

##Worsening was defined by experiencing increases in the "poor sleep quality score", and improvements as experiencing decreases in the same score.

###This score ranged from 0 to 7. Those who answered "sometimes" or "almost always" to the items "difficulty falling asleep", "awakening during night-time", "early awakening with difficulty getting back to sleep" "use of sleeping pills", "being so sleepy at daytime as to need a nap" or "not feeling rested in the morning", as well as those with an Epworth Sleepiness Scale score >10, received 1 point; their counterparts received 0 points. This score was only available in *Seniors ENRICA-2* and *Exernet*

†Information not available in EXERNET; ‡Information only available in *Seniors ENRICA-2* and *Edad con Salud*; §Information not available in *TSHA*; ‖Information only available in *Seniors ENRICA-2* and *Exernet* ¶Information only available in *Seniors-ENRICA-2*.

All models were adjusted for baseline age, sex (men or women), educational level (primary or less, secondary, or university), civil status (married, widowed, never married, divorced), housing conditions, smoking status (never, former, or current), alcohol drinking (never, former, moderate, or heavy), adherence to the Mediterranean diet (MEDAS score), body mass index (normoweight, overweight, or obese), physical activity (quartiles), hours of sleep at night (normal, short sleep, long sleep), chronic comorbidities (hypertension, diabetes, cardiovascular disease, cancer, osteo-muscular disease and depression), time since last follow-up visit, cohort of study and week of the state of alarm.

*p-value for trend <0.05
